# Transesophageal echocardiography-guided percutaneous closure of multiple muscular ventricular septal defects with pulmonary hypertension using single device: A case report

**DOI:** 10.3389/fcvm.2023.1093563

**Published:** 2023-03-23

**Authors:** Sisca Natalia Siagian, Radityo Prakoso, Brian Mendel, Zakky Hazami, Valerinna Yogibuana Swastika Putri, Zulfahmi, Damba Dwisepto Aulia Sakti, Ario Soeryo Kuncoro

**Affiliations:** ^1^ Department of Cardiology and Vascular Medicine, Division of Pediatric Cardiology and Congenital Heart Disease, National Cardiovascular Centre Harapan Kita, Universitas Indonesia, Jakarta, Indonesia; ^2^ Department of Cardiology and Vascular Medicine, Sultan Sulaiman Government Hospital, Serdang Bedagai, Indonesia; ^3^ Department of Cardiology and Vascular Medicine, National Cardiovascular Centre Harapan Kita, Universitas Indonesia, Jakarta, Indonesia; ^4^ Department of Cardiology and Vascular Medicine, Division of Non-Invasive Diagnostic and Cardiovacular Imaging, National Cardiovascular Centre Harapan Kita, Universitas Indonesia, Jakarta, Indonesia

**Keywords:** echocardiography-guided, muscular VSD, pulmonary hypertension, single device, transjugular

## Abstract

**Background:**

Surgery is typically used to correct challenging ventricular septal defects (VSDs), such as VSD with pulmonary hypertension and multiple defects. In this case report, we would like to highlight the feasibility of multiple defects VSD closure with single device percutaneously using zero-fluoroscopy technique.

**Case presentation:**

A 7-year-old child was referred with the main symptom of shortness of breath. She started experiencing repeated respiratory tract infections, feeding issues, and failure to thrive at the age of six months. Her body weight was only 18 kg. TEE revealed several muscular VSD with 2–3 mm and 12 mm diameters, 3 mm spacing between VSD, L to R shunt, AR (-), and TR mild with septal leaflet tricuspid prolapse. Following right heart catheterization (Qp:Qs 3.5, PVRi 5.23WUmsq, PVR 4.55 WU, PVR/SVR 0.16), we made the decision to correct the defect using an Amplatzer Septal Occluder (AGA) No. 16 mm using transjugular method. Full device deployment was successfully performed with several episodes of PVC storm and severe bradycardia. One and a half years after the procedure, her TVG dropped to only 18 mmHg, her visible indicators of PH subsided, and the PA dilator treatment was discontinued. Her body weight had increased to 28 kg, and she had no complaints.

**Conclusions:**

Our experience demonstrated that percutaneous closure of multiple VSD with a single device is possible, even with pulmonary hypertension.

## 1. Introduction

Surgery is typically used to correct ventricular septal defects (VSDs), particularly in challenging cases like those involving individuals with pulmonary hypertension and multiple defects. In recent years, muscular VSD found in locations that are challenging for surgeons to reach are often treated with transcatheter percutaneous closure. However, multiple defects are often repaired using multiple devices and are typically guided by fluoroscopy (1, 2). Eliminating radiation exposure during procedure is crucial since fluoroscopy effects are cumulative and raise serious issues, especially in the younger population (3). In this case report, we would like to highlight the feasibility of multiple defects VSD closure with single device using zero-fluoroscopy technique.

## 2. Case description

A 7-year-old child was referred with the main symptom of shortness of breath one year prior to admission. She started experiencing repeated respiratory tract infections, feeding issues, and failure to thrive at the age of six months, although she was not bluish. She received a pulmonary TB diagnosis and had 9 months of therapy. She continued to report having dyspnea, and an echocardiogram showed that she had multiple muscular VSDs with a left-to-right shunt and pulmonary hypertension with a total diameter of 1–1.4 cm. The results of a physical examination revealed a heart rate of 115 beats per minute, a respiratory rate of 21 breaths per minute, and a room air oxygen saturation of 98%. Her height was 122 cm, and her body weight was 18 kg. Regular first and second heart sounds were audible during auscultation, as well as a loud intensity holosystolic murmur grade 3/6 in the lower left sternal border. The patient had a D-shaped LV and severe PH with a TVG of 69 mmHg and a transVSD gradient of 15 mmHg.

**Figure 1:**
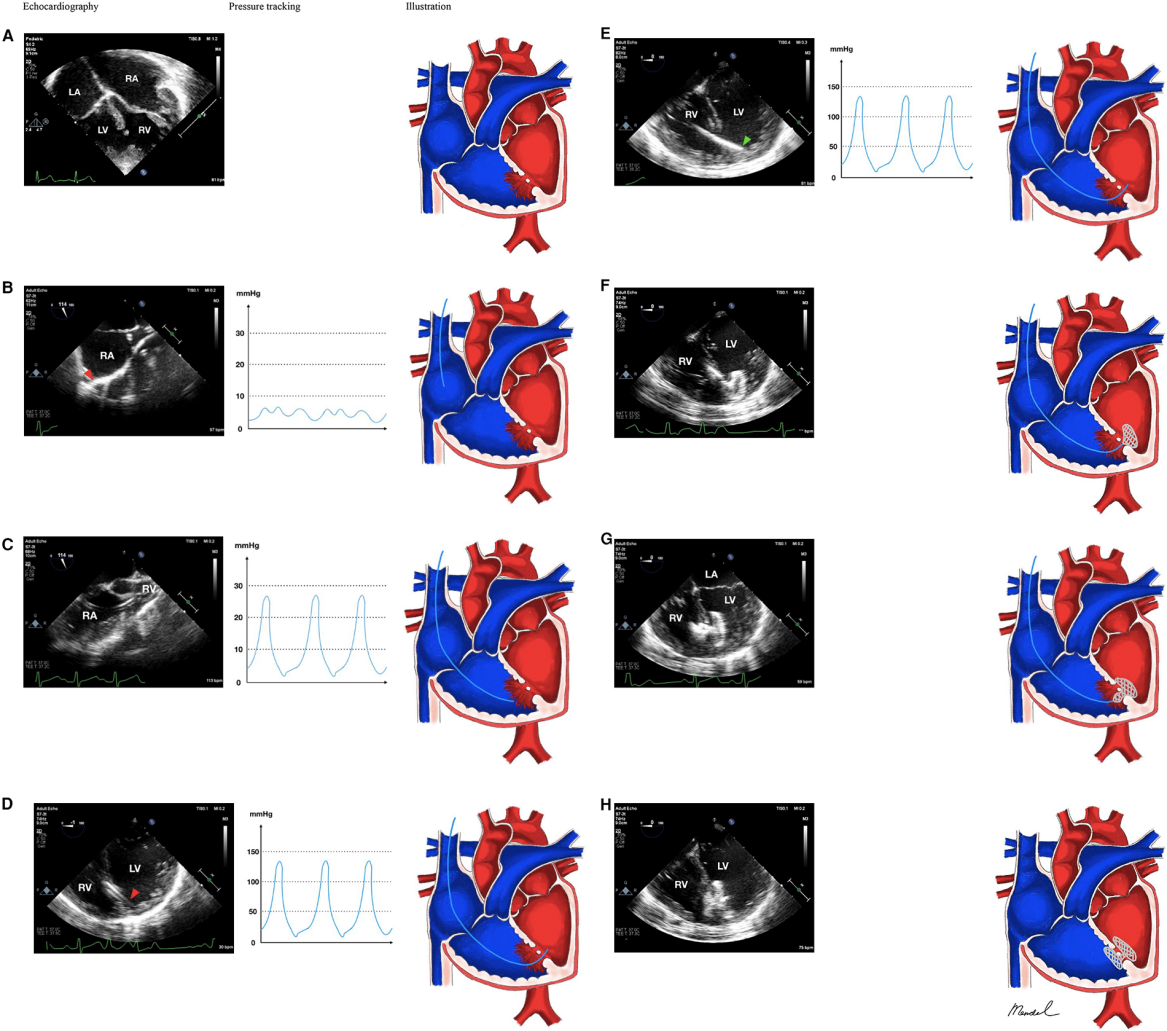
Antegrade jugular vein approach in multiple-defect muscular VSD closure with pulmonary hypertension. (**A**) Multiple VSD. (**B**) 5F sidehole Multipurpose catheter was directed from SVC towards RA. (**C**) Catheter was then directed towards RV. (**D**) The catheter successfully crossed from RV into LV. (**E**) With the assistance of 0.035″ Amplatzer stiff wire, the catheter was changed with 8F delivery sheath. (**F**) The Amplatzer device occluder (AGA) No 16 mm was delivered and one of the disc was deployed in the LV side, (**G**) and RV side. (**H**) Device stowed in place; *Red arrowhead showed the position of the catheter, green arrowhead showed the position of the delivery sheath. Notes: VSD, ventricular septal defect; SVC, superior vena cava; RV, right ventricle; LV, left ventricle; RA, right atrium; LA, left atrium*.

We chose to use the jugular vein approach to accomplish percutaneous transcatheter VSD closure. Preprocedural 98%; patient underwent general anesthesia. The patient was intubated using ETT No. 5.5% and 30% FiO2. 100% post-intubation saturation TEE revealed several muscular VSD with 2–3 mm and 12 mm diameters, 3 mm spacing between VSD, L to R shunt, AR (-), and TR mild with septal leaflet tricuspid prolapse. (Figure 1A). The decision was made to catheterize the right heart. The right femoral artery of the patient was punctured, and the patient then inserted a 4F sheath, heparin 1.000 IU, and MP sidehole 5F catheter. The right jugular vein was punctured; the MP sidehole was 5F and the sheath 6F was used. Following right cardiac catheterization (Qp:Qs 3.5, PVRi 5.23WUmsq, PVR 4.55 WU, PVR/SVR 0.16), we made the decision to correct the defect using an Amplatzer Septal Occluder (AGA) No. 16 mm using transjugular method. Through the muscular VSD, a 5F MP sidehole diagnostic catheter was introduced under the direction of transesophageal echocardiography (TEE). A 5F MP sidehole diagnostic catheter was introduced under the direction of transesophageal echocardiography (TEE) from SVC, RA, RV and LV through muscular VSD (Figures 1B–D). Using 0.035″ Amplatzer stiff wire, we change to 8F delivery sheath (Figure 1E). Full device deployment was successfully performed with several episodes of PVC storm and severe bradycardia (Figures 1F–H). Echocardiography evaluation showed no complications before, during, and after deployment of the occluder device (Figure 1H). Her symptoms and appetite steadily improved a week after the operation. She was able to engage in moderately intensive activities three months after the treatment without experiencing any discomfort.

Her TVG was 60 mmHg and 50 mmHg six months and one year after the procedure, respectively, with still visible evidence of PH. However, 1.5 years after the procedure, her TVG dropped to only 18 mmHg, her visible indicators of PH subsided, and the PA dilator treatment was discontinued. Her body weight had increased to 28 kg, and she had no complaints. The patient's ECG showed sinus rhythm during our most recent clinic visit.

## 3. Discussions

### 3.1. Management of ventricular septal defect with pulmonary hypertension

The care of patients with PAH-CHD depends heavily on behavioral change and identification of relevant risk factors. According to knowledge provided, patients with PAH-CHD who are functional class III begin treatment with the endothelin receptor antagonist bosentan (class I, level of evidence B). In contrast to placebo, bosentan significantly improved exercise capacity, haemodynamics, and functional class in the BREATHE-5 (Bosentan Randomised Trial of Endothelin Antagonist-5) trial and its long-term open label extension research, regardless of the location of septal defects (4–6).

In patients with PAH-CHD, sildenafil therapy has been demonstrated to enhance exercise capacity, Borg dyspnoea score, functional class, quality of life, and hemodynamics. When symptoms persisted despite taking the maximum amount of medication, as we did in this patient, right cardiac catheterization should be carried out to determine whether shunt closure is appropriate (4, 6).

The ESC (European Society of Cardiology) 2022 guidelines recommend shunt closure in individuals with a pulmonary-systemic flow ratio greater than 1.5:1 based on estimated pulmonary vascular resistance. When PVR is less than 3 WU and when PVR is between 3 and 5 WU, VSD closure is advised. After thorough assessment in a specialized facility, shunt closure may be taken into consideration in patients with PVR > 5 WU (6). As a result, we chose to treat on this patient's VSD.

### 3.2. Strategy for multiple ventricular septal defect closure

The apical, central, or outflow regions of the interventricular septum are the most common locations for muscular ventricular septal defects (VSDs). Up to 20% of VSDs in babies are muscular VSDs, which can have several occurrences and take on a “Swiss cheese” appearance (1, 2, 7). The VSD in our instance was situated mid-apical. In our institution, surgery is typically used as a method of multiple VSD defect repair. However, it was determined during the surgical discussion that the defect could be closed percutaneously. We are only authorized to use one device per interventional operation at our hospital due to legislation. There is no agreement on the maximum diameter at which a single device could close numerous VSD faults at the moment. For atrial septal defects, If the distance between defects is smaller than 0.5 or 0.7, only one device needs to be used (2).

As the abnormalities in the IVS are more posteriorly positioned, we attempted to operate through the jugular vein route to reduce curvature of the delivery sheath and eliminate resistance across the septum. This patient's jugular vein technique also provided a more direct route to the problem. It is also possible to approach the deficiency from the transfemoral side, although this method carries a higher risk because it is more complicated, sinuous, and difficult to traverse to the mid-apical position of the defect.

Delivered through the sheath, the multipurpose catheter and guidewire was then moved to the right ventricle *via* the tricuspid valve and readjusted with its top facing the VSD. The guidewire was gently moved and adjusted to the left ventricle *via* the VSD utilizing transesophageal echocardiography (TEE)-guided only. Since the patient was young and just 18 kg in weight, the 5F sheath expander needed to be removed gradually while the sheath was inserted into the conduit to prevent heart injury. To prevent harming nearby intracardiac structures, the sheath shouldn't be put straight and should instead be inserted about 3 to 5 cm deep.

Patients with perimembranous VSD utilize a different type of closure device than those with muscular VSD. For this patient, we utilized the Amplatzer Septal Occluder (AGA) No. 16 mm device because a sufficient size for a muscular VSD device was not available at the time of the procedure. We also chose Amplatzer Septal Occluder (AGA) since the retention disc is larger and could cover both defects with a 3 mm gap. We chose device no. 16 mm since there were two defects, and we expect that the device will cover both defects as well as the gap between them.

It is challenging to complete the closure process for a patient who has a defect adjacent to the apex since the insertion of the occluder necessitates the catheter bevel. Hemolysis that is brief and self-contained has been demonstrated by (8) Santos et al. (2018). Both perimembranous and muscular VSD that were treated with Amplatzer Septal Occluder (AGA) had a lower risk of total AV block (9); however, in our instance, some PVC storms and severe bradycardia transiently occurred after the defect closure. We suspected that several PVC storms and bradycardia episodes in our patient were caused by pulmonary hypertension rather than by the device. As we know, the most common arrhythmia complications caused by device closure are AV block and bundle branch block. We also saw no conduction abnormalities until the current follow-up, so we believe the issues were not caused by the device.

Patients with numerous VSDs frequently experience residual shunt because a proper occluder size is necessary to completely cover the defect. As a result, the type and placement of the occlusion should be carefully chosen based on the specific circumstances. After the treatment, our patient, however, displayed no symptoms of a residual shunt. Our post-procedural echocardiogram also revealed that the mitral and tricuspid valves were not damaged, as predicted when we selected and estimated the distance between the device, and the interventricular septum that would be covered by the device.

### 3.3. Feasibility of transesophageal echocardiography-guided closure

Even though ALARA (as low as reasonably achievable) principle is used, it would be preferable to completely eliminate radiation exposure risks for both the operators and the patient. In our facility, the technique would be carried out initially without any fluoroscopy, or if the echocardiography window was insufficient intraprocedurally, the technique would be converted to the standardized fluoroscopy process (1, 3, 10, 11).

As far as we know, fluoroscopy may cause certain long-term, delayed negative effects, particularly in children and newborns. Over the years, there have been more reports of skin injuries, such as redness, necrosis, and ulceration, which can be painful and disabling. There has also been an increase in the likelihood of developing neoplasms, radiation-induced cataracts, and hair loss (3).

In this instance, the finding of the VSD from the jugular approach is simple and is followed by a cross to the LV to close the defect after the patient has been intubated and is being seen with TEE. In our scenario, a fluoroscopy-guided operation is also possible, but since there are two defects, a TEE could provide a superior image. With TEE, we could instantly see the position and visualize it. Therefore, if there were multiple defects, we would prefer to advise using TEE directed. The shape and diameter of the VSD may be estimated with precision using TEE. When employing TEE, the danger of oesophageal trauma and erosion should be taken into account (1, 3, 10, 11).

## 4. Conclusions

Our experience demonstrated that percutaneous closure with a single device is possible to carry out successfully even in multiple VSD defects accompanied by pulmonary hypertension. However, the cardiac team should think about a more direct strategy to lower the danger and they should pick the patient carefully in which patient with multiple defects should be done percutaneously or surgically.

## Data Availability

The original contributions presented in the study are included in the article/Supplementary Material, further inquiries can be directed to the corresponding author/s.
